# The effect of sourdough, turnips, and butternut squash on the physicochemical and nutritional properties of Doowina functional food during fermentation

**DOI:** 10.1002/fsn3.3915

**Published:** 2024-01-02

**Authors:** Sahar Bahrami, Nafiseh Davati, Nooshin Noshirvani

**Affiliations:** ^1^ Department of Food Science and Technology, College of Food Industry Bu‐Ali Sina University Hamedan Iran; ^2^ Department of Food Science and Technology, Tuyserkan Faculty of Engineering and Natural Resources Bu‐Ali Sina University Hamedan Iran

**Keywords:** butternut squash, Doowina, functional food, sourdough, turnip

## Abstract

The dairy–cereal‐based food, known as Doowina, is one of the traditional fermented foods in Iran. We aimed to improve the health‐promoting properties of Doowina by using turnips, butternut squash, and sourdough as a new functional food with high nutritional value and antioxidant activity. Therefore, the physicochemical, microbial, and sensory properties of samples with nutritional supplements (8% turnip and 8% butternut squash) and different concentrations of sourdough (0, 0.5, and 1%) were studied during 0, 3, 6, and 9 days of fermentation time. The results showed that there was no significant difference (*p* < .05) in the moisture and ash content between the different formulations of Doowina. There was also no significant difference (*p* < .05) in the phenolic compound content and antioxidant activity of the Doowina samples during the fermentation period. However, the number of lactic acid bacteria (LAB) increased significantly (*p* < .05) until the 6th day of fermentation, and the protein content decreased significantly (*p* < .05) in all samples during the fermentation period. According to the results, the samples with butternut squash and sourdough had the highest total phenolic content, the highest antioxidant activity, the highest linoleic acid content, and the highest sensory rating of all samples.

## Graphical abstract

Nowadays, there is a strong tendency to use functional foods such as fermented foods based cereal and dairy products. The addition of sourdough, turnips, and butternut squash improves the nutritional value and healthy properties of Doowina during fermentation. Doowina with butternut squash and 0.5% sourdough is presented as a new functional dairy–cereal food rich in unsaturated fatty acids and antioxidant compounds.

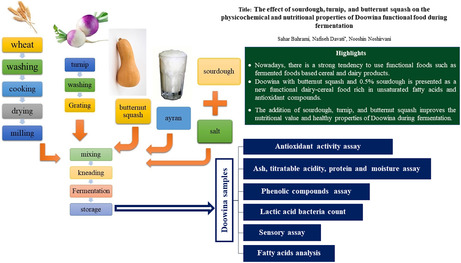

## 1 INTRODUCTION

Recently, the increasing consumption of functional foods such as fermented products has significantly reduced the risk of diseases and improved consumer health (Ahn‐Jarvis et al., 2013; Teleky et al., 2022). The origin of fermented products can be traced back to the Middle East (Mashak et al., 2014). The fermentation process, as a food preservation technique, converts the raw materials into healthy and nutritious products with better digestibility. The microorganisms, especially lactic acid bacteria (LAB), which are transferred from dairy products and vegetables to the fermentation containers, produce antimicrobial metabolites and organic acids, which can lead to a longer shelf life by inhibiting the growth of unwanted microbial flora (Leroy & De Vuyst, 2004; Steinkraus, 2018; Zapaśnik et al., 2022). Fermented dairy–cereal‐based foods can provide valuable bioactive compounds that contain sufficient essential amino acids due to the presence of milk protein, which compensates for the deficiency of essential amino acids such as lysine and threonine in cereals and promotes consumer health (Mashak et al., 2014). Doowina in Kurdish or Tarkhineh, Tarkhowana, Tarhana in Turkish, one of the local products of the western regions of Iran (Kermanshah, Ilam, Kurdistan, Lorestan, and Hamedan), is made from the fermentation of wheat flour, salt, and concentrated ayran (drinking yogurt) (Moradi et al., 2022). It tastes like sour wheat bread. This food can be a good source of organic acids, minerals, free amino acids, vitamins, ascorbic acid, and folic acid (Bilgiçli, Elgün, Herken, et al., 2006; Bilgiçli, Elgün, & Türker, 2006). In addition, the probiotic properties of this food and its health effects, such as lowering cholesterol levels, regulating blood glucose levels in diabetics, and reducing the risk of cancer in consumers, have already been mentioned (Erkan et al., 2006). In addition to the intrinsic microbial flora of the raw materials, sourdough can also be used to intensify the fermentation of Doowina. Sourdough contains LAB and yeasts, with LAB being particularly beneficial for the nutritional and functional properties of sourdough fermentation. LAB contributes to the increase in nutritional value, soluble fiber, and phenolic compounds, while yeasts are responsible for aroma formation and leavening, which improve the sensory properties of fermented products (Curiel et al., 2015). Sourdough fermentation, which is promoted by the activity of LAB, can improve the content of bioactive substances, sensory properties, texture, and nutritional value of foods made from cereals and legumes. It can also improve mineral absorption, lower the glycemic index, and reduce the presence of antinutritive factors such as phytic acid and raffinose (Coda et al., 2010, 2011, 2014; Gabriele et al., 2019; Moroni et al., 2012). Several studies have reported that sourdough improves the nutritional quality of wheat‐based products as follows: it increases the content of *fatty acid* amides (FAAs), free essential amino acids, and γ‐aminobutyric acid (GABA); decreases the starch hydrolysis index (HI) (Coda et al., 2010); improves the protein efficiency ratio (PER), net protein ratio (NPR), apparent digestibility (AD), and actual digestibility (Peñaloza‐Espinosa et al., 2011); decreases trypsin inhibitor activity; increases protein digestibility (Bartkiene et al., 2011); improves antihypertensive, antioxidant, and anti‐inflammatory properties; and increases phytase activity (Gabriele et al., 2023; Rizzello et al., 2014). Turnip (*Brassica rapa* ssp. Rapa) has been used as medicine and food since ancient times. It is rich in vitamins, minerals, anthocyanins, flavonoids, omega‐3 fatty acids, fiber, proteins, and biologically active compounds (Scalzo et al., 2008). The antimicrobial, anticancer, anti‐hypoxia, anti‐diabetic, antiviral, and antioxidant activities of turnips have been mentioned in previous studies (Bochkov et al., 2012; Bradley, 2005; Cao et al., 2021). Butternut squash (*C. moschata*) is another valuable source of vitamins, fiber, β‐carotene, tocopherol, minerals, and bioactive substances (Armesto et al., 2020). Therefore, it is recommended that turnips and butternut squash be consumed as valuable vegetables for the preparation of functional foods (Auger et al., 2010; Francisco et al., 2009; Javed et al., 2019). It is expected that Doowina prepared with wheat, ayran, sourdough, turnips, and butternut squash can be rich in micronutrients and antioxidants. In addition, the presence of various organic acids and the low moisture content provide a bacteriostatic effect that increases the shelf life of Doowina (Ozdemir et al., 2007). Due to the increase of cancer and other diseases in human society, improving the nutritional value of Doowina as a staple food by changing the formulation can help maintain the health of consumers. It should be noted that there are very few studies on Doowina, and as far as we know, no studies have been conducted on the nutritional fortification of Doowina with turnips, butternut squash, and sourdough. In addition, Doowina has a dominant cereal taste, which can be improved by enriching it with turnips and butternut squash. Nowadays, nutritious supplements such as fruits and vegetables (turnips and butternut squash) can be used to improve the sensory properties and nutritional value of many cereal products. For example, Haș et al. (2023) enriched a millet‐ and oat‐based snack with freeze‐dried elderberry powder to access bioactive compounds and vital nutrients. To increase the nutritional value of Doowina and improve its sensory properties, this study investigated the changes in physicochemical properties, antioxidant activity, unsaturated fatty acid (UFA) content, LAB community, and sensory properties of different formulations of Doowina with turnips and butternut squash, whose fermentation process was intensified by increasing the proportion of sourdough.

## 2 MATERIALS AND METHODS

All chemical compounds, solvents, and reagents were prepared by Merck Co., Darmstadt, Germany. Wheat groats, ayran, salt, sourdough, butternut squash, and turnips were purchased in the local market of Hamedan, a province in western Iran.

### 2.1 Doowina preparation

**FIGURE 1:**
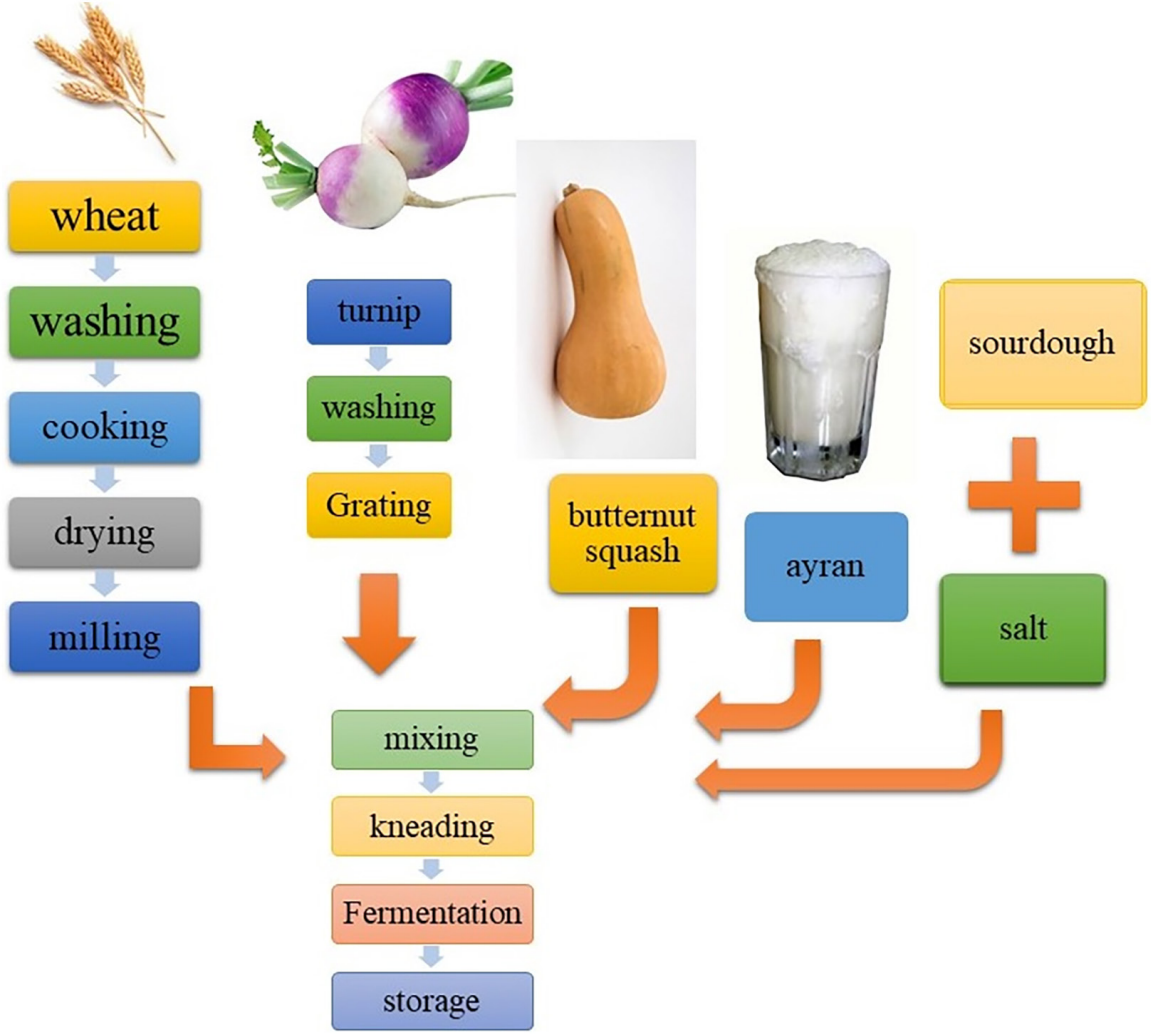
Flowchart of Doowina production.

All Doowina samples were prepared according to a traditional recipe in the autumn, which is shown in Figure 1. First, the wheat groats were cleaned, washed, and cooked at 80°C for 1 h, followed by drying in the oven at 60°C until the constant dw (dry weight). The dried groats (24.5%) were ground and then mixed well with ayran (70.2%) and salt (5.3%). After that, nutritious supplements, including butternut squash and turnips, were added to the primary mixture (P) according to Table 1. Then, all the ingredients were kneaded for 5 min and placed in a sieve to drain the excess water. The prepared samples were filled into sterile jars and fermented spontaneously at room temperature (20 ± 2°C) for 9 days. The Doowina was aseptically sampled in triplicate every 3 days during fermentation for further analysis.

**TABLE 1:** Different formulations for Doowina samples.

Formulation code	Ingredients % (wet b, w/w)
P	Primary mixture
P.T	Primary mixture + turnip (8%)
P.Sq	Primary mixture + butternut squash (8%)
P.S1	Primary mixture + sourdough (0.5%)
P.S1.T	Primary mixture + sourdough (0.5%) + turnip (8%)
P.S1.Sq	Primary mixture + sourdough (0.5%) + butternut squash (8%)
P.S2	Primary mixture + sourdough (1%)
P.S2.T	Primary mixture + sourdough (1%) + turnip (8%)
P.S2.Sq	Primary mixture + sourdough (1%) + butternut squash (8%)

### 2.2 Preparation of an extract from Doowina

Each sample (0.3 g) was mixed with 10 mL of 70% aqueous acetone and shaken in a shaking incubator (Parsian Teb, Co, Iran) at 60 rpm for 30 min (20 ± 2°C). The extracts were centrifuged (Universal 320 R, Hetich Co, Germany; 15,000 × *g*, 20 min, 20 ± 2°C), and the supernatant was collected for further analysis (O'Callaghan et al., 2019).

### 2.3 Physicochemical properties of the Doowina samples during fermentation

The ash and moisture content of the Doowina samples were measured during fermentation, according to the Association of Official Analytical Chemists (AOAC, 2000). The proteins of the Doowina extracts were measured by the Bradford method (Bradford, 1976) using bovine serum albumin (BSA) as a standard, assuming it to be 100% pure. To measure the titratable acidity (TA) of Doowina, 10 g of each sample was mixed with 40 mL of 67% (v/v) ethanol, then filtered (Whatman No. 30) and titrated with NaOH (0.1 N) in the presence of phenolphthalein as an indicator (Xiong et al., 2012).

### 2.4 Fatty acid profile analysis of Doowina samples during fermentation

The oil from Doowina samples was extracted according to Bligh and Dyer (1959); Bochkov et al. (2012). The esterification of the fatty acids was carried out by transesterification with potassium hydroxide according to ISO 5509:2000 (ISO, 2000), with minor modifications. Initially, 1 g of the extracted oil was homogenized with 7 mL of n‐hexane and 3 mL of saturated methanolic KOH solution. The mixture was shaken and heated at 49°C for 10 min. The upper organic layer was used as a methyl ester source for gas chromatography (GC) analysis. The fatty acid methyl esters were analyzed using a GC (Shimadzu 2014, Japan) with a split/splitless injector (split mode). The analytes were injected into the SGE‐BPX70 capillary column (60 m × 0.25 mm × 0.25 μm) via 1‐μl split injections with a split ratio of 50:1. The temperature of the injection port was held at 250°C and the FID temperature was set to 260°C. The oven temperature program was as follows: hold at 100°C for 2 min; heat from 100 to 220°C at a rate of 6°C/min for 8 min; and hold at 220°C until the end of the test; the carrier gas was N_2_ at a flow rate of 0.87 mL/min. The fatty acid methyl esters were identified by their retention times compared to the reference standards and determined as a percentage (%) of the total fatty acids in each sample. The fatty acid profiles of the different formulations of Doowina during fermentation were analyzed in a replicate.

### 2.5 Total polyphenolic content of Doowina samples during fermentation

The Folin–Ciocalteu assay (Singleton et al., 1999) was used to determine the total phenolic content of the Doowina extracts. For this purpose, 1 mL of Folin–Ciocalteu reagent was mixed with 1 mL of each Doowina extract, and then 1 mL of 20% Na_2_CO_3_ was added. The mixtures were then left in the dark at room temperature for 30 min. The absorbance of the samples was determined using a spectrophotometer model UV/VIS XD 7500 (Lovibond, Germany) at 765 nm. Gallic acid and a sample containing water and reagent were used as standards and blanks, respectively. The results were expressed as mg gallic acid/mL of extract.

### 2.6 Antioxidant activity of Doowina samples during fermentation

The antioxidant activity was determined by the free radical scavenging activity of 2,2‐diphenyl‐1‐picrylhydrazyl (DPPH) according to the method described by Brand‐Williams et al. (1995). First, 300 μL of each Doowina extract was mixed with 2700 μL of a methanolic DPPH solution (0.06 mM). The mixtures were vortexed and kept at room temperature in a dark place for 1 h. The absorbance of the solutions was then determined using a spectrophotometer model UV/VIS XD 7500 spectrophotometer (Lovibond, Germany) at 515 nm. The control sample, containing all reagents except the test compound, was applied. The percentage of inhibition was determined using Equation (1).

(1) $$\text{DPPH scavenging activity}\%=\frac{\text{Control absorbance}-\text{Sample absorbance}}{\text{Control absorbance}}\times 100$$

### 2.7 LAB community changes in Doowina samples during fermentation

For the identification of LAB, 1 g of each sample was added to 9 mL of sterile Ringer, and then serial dilutions up to 10^−8^ were prepared. The corresponding dilutions were plated on de Man, Rogosa, and Sharpe Agar (MRS; Merck, Germany) and the plates were incubated at 37°C under anaerobic conditions with a gas pack (Anaerocult® A; Merck, Darmstadt, Germany) for 48 h (Pommerville, 2007).

### 2.8 Sensory analysis of Doowina samples

The sensory properties of the Doowina samples were evaluated at the end of the fermentation period. Samples were evaluated for color, taste, texture, and overall acceptability on a 5‐point hedonic scale (1 = very poor, 2 = poor, 3 = fair, 4 = good, 5 = excellent).

### 2.9 Statistical analysis

Experimental data were analyzed by ANOVA (*p* < .05) using SPSS version 16, and significant differences between means were determined using Duncan's multiple‐range test.

## 3 RESULTS AND DISCUSSION

### 3.1 Changes in physicochemical properties

As shown in Table 2, there was no significant difference (*p* < .05) in the moisture content of the different Doowina formulations. The moisture content decreased in all samples during the fermentation period. The highest moisture content (25.18%) was obtained for P.T (with 8% turnips) on day 0 of fermentation, while the lowest moisture content (23.36%) was obtained for P.S2 (with 1% sourdough) on the 9th day of fermentation. The results showed that the addition of sourdough reduced the moisture content; however, the addition of nutrient supplements increased the moisture content compared to the control sample. The reduction in moisture content in the sample containing sourdough is attributed to the water consumption of the yeasts and LAB due to their metabolism. In a study, Gurbuz et al. (2010) reported that the moisture content of Tarhana does not change during the fermentation period compared to the control sample, which is not consistent with the results of the present study. Another reason for the decrease in moisture content in our study can be attributed to the evaporation of water during the storage period. The reason for the increase in moisture content in the samples to which butternut squash and turnips were added could be due to the high fiber content and high water retention capacity of the added vegetables, which increase the moisture content of the sample compared to the control sample (Zomorod et al., 2020). As shown in Table 2, there was no considerable difference in ash content between the different formulations. The ash content increased in all samples during the fermentation period. Furthermore, the results showed that the addition of sourdough and nutritive supplements increased the ash content of the samples compared to the control sample. The highest ash content (5.95%) was found for P.S2.Sq (sample with butternut squash and 1% sourdough) on the 9th day of fermentation, while the lowest ash content (2.80%) was found for the control sample on the 0th day of fermentation. The results of the present study are consistent with the results of Zomorod et al. (2020) and Gurbuz et al. (2010). As shown in Table 2, the TA value increased in all samples during the fermentation period. There was no significant difference (*p* < .05) in TA between the different formulations of Doowina at the beginning of fermentation. However, there was a significant difference (*p* < .05) in TA between the different formulations at the end of fermentation. The highest TA (7.80%) was obtained for P.S2.Sq (sample with 8% butternut squash and 1% sourdough) on the 9th day of fermentation, while the lowest TA (1.58%) was obtained for the control sample on the 0th day of fermentation. The increase in TA may be mainly due to the fermentation of carbohydrates and the formation of organic acids by LAB in sourdough and ayran (Erbaş et al., 2005). Higher amounts of TA in the samples containing turnips and butternut squash can be attributed to a higher carbohydrate content for fermentation compared to the other samples, which subsequently significantly increased TA with the addition of sourdough (LAB). Similar reports were also made by Erbaş et al. (2005), Kivanc and Funda (2017), and Hassan and Gadallah (2018) for Tarhana.

**TABLE 2:** Effect of formulation and fermentation time on the physicochemical properties (%) of the Doowina sample during fermentation.

	Days	P	P.T	P.Sq	P.S1	P.S1.T	P.S1.Sq	P.S2	P.S2.T	P.S2.Sq
Moisture	0	25.05 ± 0.7^a^	25.18 ± 0.4^a^	25.10 ± 0.6^a^	25.00 ± 0.3^a^	25.05 ± 0.2^a^	25.03 ± 0.4^a^	24.86 ± 0.2^a^	25.00 ± 0.5^a^	24.93 ± 0.8^a^
	3	24.55 ± 0.1^a^	24.81 ± 0.1^a^	24.70 ± 0.2^a^	24.51 ± 0.1^a^	24.57 ± 0.3^a^	24.54 ± 0.1^a^	24.43 ± 0.6^a^	24.47 ± 0.7^a^	24.47 ± 0.2^a^
	6	24.23 ± 0.2^a^	24.47 ± 0.1^a^	24.43 ± 0.2^a^	24.10 ± 0.5^a^	24.17 ± 0.1^a^	24.17 ± 0.2^a^	24.00 ± 0.4^a^	24.07 ± 0.4^a^	24.05 ± 0.1^a^
	9	23.60 ± 0.1^a^	23.68 ± 0.3^a^	23.63 ± 0.1^a^	23.50 ± 0.1^a^	23.55 ± 0.7^a^	23.53 ± 0.5^a^	23.36 ± 0.1^a^	23.50 ± 0.4^a^	23.43 ± 0.1^a^
Ash	0	2.8 ± 0.08^b^	2.86 ± 0.02^b^	2.93 ± 0.01^b^	2.84 ± 0.03^b^	3.06 ± 0.08^b^	3.16 ± 0.01^a,b^	3.23 ± 0.02^a,b^	3.5 ± 0.01^a^	3.63 ± 0.05^a^
	3	3.03 ± 0.04^b^	3.1 ± 0.02^b^	3.2 ± 0.02^a,b^	3.13 ± 0.01^a,b^	3.13 ± 0.07^a,b^	3.26 ± 0.07^a,b^	3.33 ± 0.01^a,b^	3.5 ± 0.04^a^	3.96 ± 0.02^a^
	6	4.15 ± 0.02^c^	4.22 ± 0.01^b,c^	4.32 ± 0.07^b,c^	4.31 ± 0.05^b,c^	4.31 ± 0.04^b,c^	4.44 ± 0.05^b,c^	4.56 ± 0.04^b^	4.73 ± 0.01^b^	5.19 ± 0.08^a^
	9	4.68 ± 0.02^d,e^	4.75 ± 0.04^d,e^	4.85 ± 0.05^d^	4.95 ± 0.02^c,d^	4.95 ± 0.02^c,d^	5.08 ± 0.09^c^	5.32 ± 0.02^b,c^	5.49 ± 0.06^b^	5.95 ± 0.05^a^
TA	0	1.58 ± 0.05^a^	1.61 ± 0.02^a^	1.61 ± 0.03^a^	1.65 ± 0.04^a^	1.67 ± 0.04^a^	1.67 ± 0.03^a^	1.72 ± 0.05^a^	1.74 ± 0.05^a^	1.74 ± 0.08^a^
	3	2.61 ± 0.02^c^	2.72 ± 0.01^b,c^	2.77 ± 0.01^b,c^	2.80 ± 0.02^b^	2.83 ± 0.05^b^	2.91 ± 0.08^a,b^	2.96 ± 0.07^a,b^	3.01 ± 0.04^a,b^	3.10 ± 0.12^a^
	6	4.19 ± 0.1^d^	4.41 ± 0.14^c,d^	4.52 ± 0.11^c^	5.17 ± 0.14^b,c^	5.23 ± 0.9^b^	5.32 ± 0.1^b,c^	5.52 ± 0.1^a^	5.56 ± 0.1^a^	5.66 ± 0.15^a^
	9	6.10 ± 0.1^f^	6.23 ± 0.15^e,f^	6.53 ± 0.12^e^	6.89 ± 0.07^d^	7.11 ± 0.12^c^	7.38 ± 0.11^b,c^	7.47 ± 0.13^b^	7.57 ± 0.16^a,b^	7.80 ± 0.11^a^

*Note*: Different letters indicate statistically significant differences at *p* < .05 in each row.

Abbreviations: P, primary mixture; P.S1, primary mixture + sourdough (0.5%); P.S1.Sq, primary mixture + sourdough (0.5%)  + butternut squash (8%); P.S1.T, primary mixture + sourdough (0.5%) + turnip (8%); P.S2, primary mixture + sourdough (1%); P.S2.Sq, primary mixture + sourdough (1%) + butternut squash (8%); P.S2.T, primary mixture + sourdough (1%) + turnip (8%); P.Sq, primary mixture + butternut squash (8%); P.T, primary mixture + turnip (8%).

### 3.2 Changes in protein content

**FIGURE 2:**
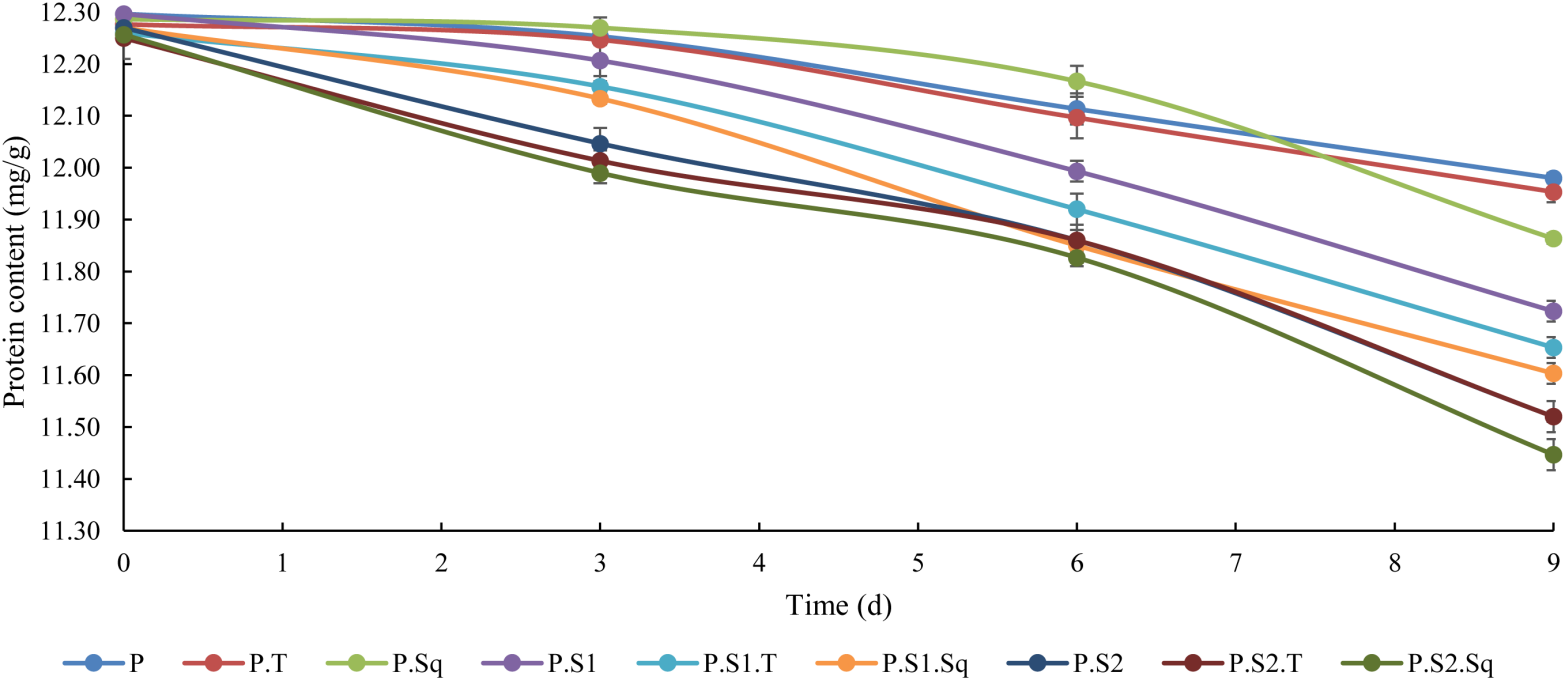
Effect of formulation and fermentation time on the protein content in Doowina. Vertical bars represent the standard deviation (*n* = 3). P, primary mixture; P.T, primary mixture + turnip (8%); P.Sq, primary mixture + butternut squash (8%); P.S1, primary mixture + sourdough (0.5%); P.S1.T, primary mixture + sourdough (0.5%) + turnip (8%); P.S1.Sq, primary mixture + sourdough (0.5%) + butternut squash (8%); P.S2, primary mixture + sourdough (1%); P.S2.T, primary mixture + sourdough (1%) + turnip (8%); P.S2.Sq, primary mixture + sourdough (1%) + butternut squash (8%).

The protein content of the different Doowina formulations over time is shown in Figure 2. According to the results, the protein content decreased significantly (*p* < .05) in all samples during the fermentation period. It should be noted that the effect of sourdough on the reduction of protein content during the fermentation period was significant (*p* < .05), while supplementation alone, especially turnip, showed no significant effect on the protein of Doowina. Meanwhile, the simultaneous effect of nutrient supplements and sourdough during the fermentation period on the amount of protein was significant (*p* < .05). The comparison of the protein content of Doowina at different concentrations of sourdough showed that the addition of sourdough during fermentation led to a reduction in the protein content of Doowina. The comparison of the samples on days 0, 3, 6, and 9 of fermentation also showed that an increase in fermentation time caused a decrease in protein content (*p* < .05). Thus, the highest amount of protein was recorded in the control sample (11.98 mg/g) and the lowest amount in P.S2.Sq (11.45 mg/g) at the end of the fermentation time. Doowina contains milk proteins (casein, globulin, and albumin) and wheat proteins (glutenin and gliadin) derived from the ayran and wheat flour used for the production of Doowina. Therefore, the amount of protein in Doowina is directly related to the amount of ayran and wheat flour in the formulation (Mohammadi & Ostovar, 2023). As shown in Figure 2, the protein content of Doowina varied between 11.45 and 12.30 mg/g during fermentation. As can be seen in Figure 2, the addition of butternut squash and turnips alone had no significant effect on the amount of protein. The results are consistent with those of Ghafoori and Hosseini Ghaboos (2018), who indicated that the addition of butternut squash powder had no significant effect on the change in protein content (*p* < .05) in breakfast cereals fortified with butternut squash powder. According to the results, the addition of yeast to the samples reduced the protein content. This protein degradation could be due to the proteolytic activities of LAB and yeasts (Çelik et al., 2005). Previous reports have shown that yeasts and LAB can alter the protein content of fermented foods and increase the levels of total nitrogen, hypotensive γ‐aminobutyric acid (GABA), and essential amino acids (Balasubramanian et al., 2015; Coda et al., 2015; Khattab & Arntfield, 2009; Palanisamy et al., 2012; Soni et al., 1985). It has also been reported that *Saccharomyces cerevisiae* can alter protein content during fermentation and improve protein quality (Khattab et al., 2009). Therefore, the protein content of Doowina decreased due to the degradation process by the microbial flora and was probably converted into valuable nutrients. Our results are consistent with the findings of Gurbuz et al. (2010) and Soyuçok et al. (2021).

### 3.3 Changes in phenolic compounds

**FIGURE 3:**
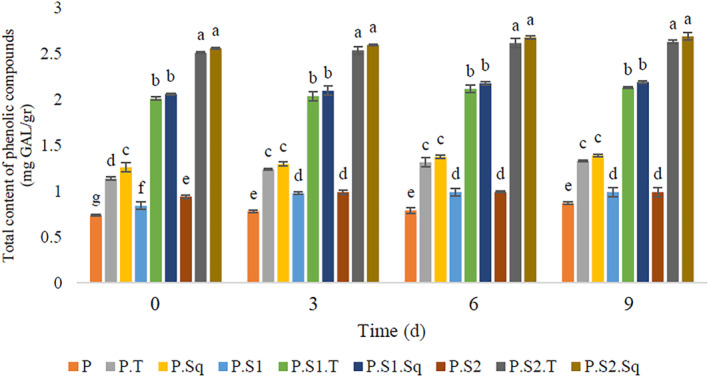
Effect of formulation and fermentation time on the total content of phenolic compounds in Doowina. Vertical bars represent the standard deviation (*n* = 3). Different letters indicate statistically significant differences (*p* < .05). P, primary mixture; P.T, primary mixture + turnip (8%); P.Sq, primary mixture + butternut squash (8%); P.S1, primary mixture + sourdough (0.5%); P.S1.T, primary mixture + sourdough (0.5%) + turnip (8%); P.S1.Sq, primary mixture + sourdough (0.5%) + butternut squash (8%); P.S2, primary mixture + sourdough (1%); P.S2.T, primary mixture + sourdough (1%) + turnip (8%); P.S2.Sq, primary mixture + sourdough (1%) + butternut squash (8%).

Nowadays, the consumption of foods containing phenolic compounds as potential antioxidants is strongly recommended to prevent diseases related to oxidative stress (Morton et al., 2000; Newmark, 1996; Sharma et al., 2011). The changes in the content of phenolic compounds for different formulations of Doowina during fermentation are shown in Figure 3. The results show that increasing the sourdough percentage from 0 to 1% has a significant effect (*p* < .05) on the content of phenolic compounds, in the different samples of Doowina. However, there was no significant difference (*p* < .05) in the content of phenolic compounds in the Doowina samples during the fermentation period. In addition, the Doowina samples with sourdough (1%) together with butternut squash or turnip had the highest content of phenolic compounds including 2.69 and 2.63 (mg GAL/g), respectively. This result is in line with the findings of Torino et al. (2013) and Limón et al. (2015). According to the results, the control formulation without butternut squash or turnip had lower phenolic compounds than the formulations with butternut squash or turnip. In addition, the phenolic compound levels in the samples containing turnips and butternut squash increased in the presence of sourdough. This could be due to the release of phenolic compounds by the enzymatic activities of the microbial flora of Doowina, especially sourdough (Adebo & Gabriela Medina‐Meza, 2020; Omedi et al., 2019). Based on our results, increasing the content of phenolic compounds in these Doowina formulations during fermentation may improve the health benefits of this product (Adebo & Gabriela Medina‐Meza, 2020). According to previous reports, the content of phenolic compounds may change as a result of plant fermentation (Bartolomé et al., 1997; Dueñas et al., 2005). Several reports suggest that LAB play a key role in altering the content of phenolic compounds due to esterase and glycosidase activities leading to the production of free phenolic acids and aglycones (Esteban‐Torres et al., 2015; Ferreira et al., 2013; Jiménez et al., 2014; Limón et al., 2015). In addition, the degradation of the wall structure of plant cells by microbial activities can lead to the release of phenolic compounds and the subsequent synthesis of various bioactive compounds (Đorđević et al., 2010; Katina et al., 2007). Turnips and butternut squash are suggested as excellent sources of phenolic compounds for the production of Doowina.

### 3.4 Changes in antioxidant activity

**FIGURE 4:**
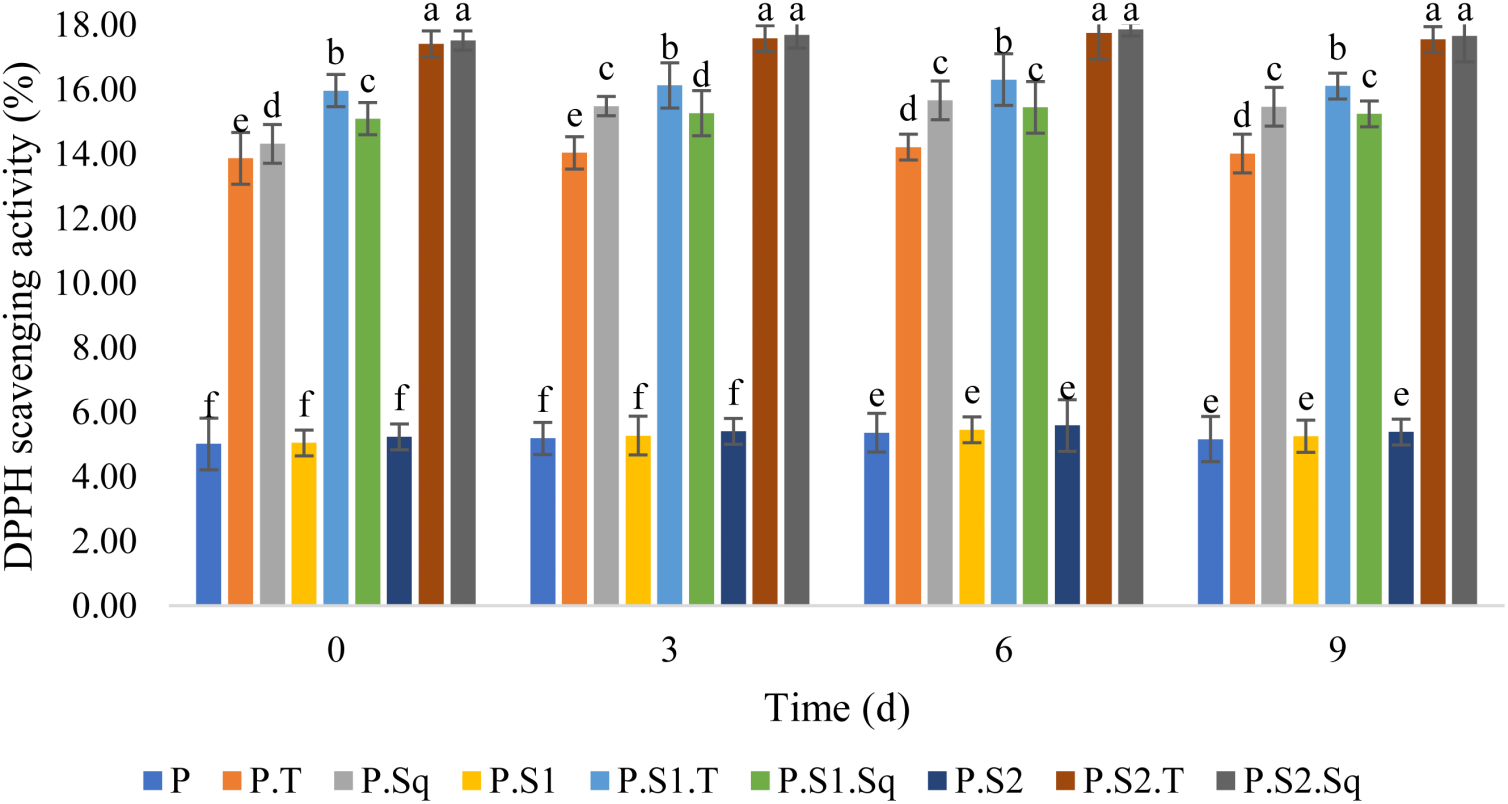
Effect of formulation and fermentation time on the antioxidant activity in Doowina. Vertical bars represent the standard deviation (*n* = 3). Different letters indicate statistically significant differences (*p* < .05). P, primary mixture; P.T, primary mixture + turnip (8%); P.Sq, primary mixture + butternut squash (8%); P.S1, primary mixture + sourdough (0.5%); P.S1.T, primary mixture + sourdough (0.5%) + turnip (8%); P.S1.Sq, primary mixture + sourdough (0.5%) + butternut squash (8%); P.S2, primary mixture + sourdough (1%); P.S2.T, primary mixture + sourdough (1%) + turnip (8%); P.S2.Sq, primary mixture + sourdough (1%) + butternut squash (8%).

According to Figure 4, there was no significant difference (*p* < .05) in the antioxidant activity of the Doowina samples containing only sourdough (0.5 and 1%) and the control sample, while the antioxidant activity differed significantly (*p* < .05) between the formulations of the Doowina samples containing only nutritional supplements and the samples containing nutritional supplements together with sourdough (0.5%). However, there was no significant difference (*p* < .05) in the antioxidant activity of the Doowina samples during the fermentation period. In all Doowina samples, the antioxidant activity increased slightly until the 6th day of fermentation and then remained approximately constant until the 9th day of fermentation. Similar to the results of the phenolic compounds analysis, the Doowina samples containing turnips or especially butternut squash in the presence of sourdough showed higher antioxidant activity than the others, so that the highest antioxidant activity of the Doowina sample with sourdough (1%) together with butternut squash (8%) was observed on the 6th day of fermentation. Fruits and vegetables contain antioxidant compounds such as phenolic acids, flavonoids, anthocyanins, tannins, lignans, and catechins. Several reports indicate that phenolic compounds have high antioxidant activity (Frias et al., 2016), and numerous phenolic compounds with antioxidant activity are biosynthesized by microorganisms due to their phenolic acid decarboxylase and reductase activities (María Landete et al., 2015; Rodríguez et al., 2009). Among the microbial communities involved in the fermentation process, the LAB of sourdough may be mainly responsible for increasing antioxidant activity by releasing freely soluble polyphenolic compounds (Frias et al., 2016). The microbial enzymes in sourdough, such as glucosidase, amylase, cellulase, inulinase, phytase, xylanase, tannase, esterase, invertase, and lipase, destroy the plant cell walls and subsequently ensure the extraction of flavonoids, which leads to an increase in phenolic compounds with antioxidant activity. These enzymes can degrade phenolic glycoside bands and consequently release aglycones (Martins et al., 2011). Structural changes in phytochemicals during fermentation may be another factor that increases the antioxidant activity of fermented plant products (Othman et al., 2009). Similarly, Gocmen et al. (2004) and Tarakci et al. (2013) reported that the addition of polyphenolic‐containing plants to Tarhana increased the antioxidant activity of fermented products. The reduction in antioxidant activity in the final stage of fermentation may be due to a decrease in the LAB population due to nutrient depletion by yeasts in the sourdough (Gurbuz et al., 2010).

### 3.5 Changes in fatty acid profile

The fatty acid profile can influence the physicochemical, sensory, and nutritional properties of fermented products. The results of the fatty acid profile of Doowina were classified into three groups: short‐chain fatty acids, saturated fatty acids, and UFAs (see Table 3 and Data S1). The results show that saturated fatty acids dominate in all different Doowina formulations. In all samples, saturated fatty acids accounted for about 80% of the total fatty acids, followed by UFAs. The short‐chain fatty acids had the lowest value of all. Among the short‐chain fatty acids, butyric acid (C4:0), *caproic* acid (C6:0), and caprylic acid (C8:0) were the most abundant in the different formulations of Doowina. This could be due to the formation of free butyric acid during lactic acid fermentation and the enzymatic conversion of amino acids into butyric acid (Erbaş et al., 2006). According to the results, the addition of turnips and butternut squash increased the short‐chain fatty acids compared to the control sample. Moreover, as the sourdough concentration increased, the percentage of unsaturated and short‐chain fatty acids, as valuable fatty acids, increased in Doowina. The results showed that increasing the fermentation time from day 0 to day 6 led to an increase in short‐chain fatty acids in all samples. However, from the 6th to the 9th day of fermentation, the content of fatty acids decreased. The reason for the increase in short‐chain fatty acids in Doowina until the 6th day could be due to the degradation of fatty acids by bacterial lipolytic activity and the production of short‐chain fatty acids. In the triglycerides of dairy products, the short‐chain fatty acids are mainly esterified at the sn‐3 position. Lipolysis readily releases the fatty acids from the sn‐1 and sn‐3 positions of the mono‐, di‐, and triglycerides, leading to an increase in short‐chain fatty acids (Michalski, 2009). The highest percentage of short‐chain fatty acids was observed in the P.S2.Sq formulation (9.28%) on the 6th day and the lowest in the control sample (4.93%) on the 9th day of fermentation. Table 3 shows that saturated fatty acids were highest on day 0 of fermentation in all formulations, and their content decreased with increasing fermentation time. However, by the end of the fermentation period, their content was always higher than that of other fatty acids. Most of the saturated fatty acids found in Doowina were palmitic acid (C16:0; 28.73–33.56%), followed by stearic acid (C18:0; 4.54–9.0%), which probably originated from buttermilk (Data S1). Erbaş et al. (2006) found that the main source of palmitic acid in Tarhana is yogurt. Sumarmono and Sulistyowati (2015) presented myristic, stearic, and palmitic acids as the main fatty acids in yogurt prepared with three different types of milk. The addition of turnips and butternut squash, with or without sourdough, led to a reduction in saturated fatty acids. In general, LAB contributes relatively little to lipolysis, but other microbial flora of animal and plant origin, e.g. yeasts, often have high lipolytic activity. The highest percentage of saturated fatty acids was observed in the control formulation on day 0 (62.19%) and the lowest in the P.S2.Sq formulation on the 9th day of fermentation (57.06%). The predominant UFA in each of the Doowina samples was oleic acid (C18:1; 15.53–21.85%), followed by linoleic acid (C18:2; 15.10–29.50%; Data S1). Similar results were observed in the data reported by Ovando‐Martinez et al. (2014) and Mohammadi and Ostovar (2023) in the samples of the Tarhana study. The presence of linoleic acid, one of the essential fatty acids in Doowina, was described by Erbaş et al. (2006) and Ovando‐Martinez et al. (2014). Higher levels of linoleic acid are mainly found in the Doowina samples with butternut squash at the end of the fermentation period. Butternut squash has a linoleic acid content of about 30% of the total fatty acid content, which leads to a higher linoleic acid content in the samples with butternut squash (17.17–29.5%). Linoleic and linolenic acids are considered essential as they cannot be synthesized by the human body and must be achieved through food. A sufficient dietary intake of essential fatty acids (linoleic acid and α‐linolenic acid) is necessary for the optimal functioning of the immune system and the brain (Chang et al., 2009). According to Gabrial et al. (2010), consumption of Tarhana reduced low‐density lipoprotein (LDL, bad blood cholesterol) and triglycerides and increased high‐density lipoprotein (HDL, good blood cholesterol). Prinyawiwatkul et al. (1996) reported that the lipid content in cowpea‐like *tempeh* increased after 24 h of mushroom fermentation (from 2.2% to 2.8%), and the major UFAs were linolenic acid and linoleic acid. Niveditha et al. (2012) indicated that the fermentation process by *Rhizopus oligosporus* resulted in a slight decrease in the lipid content of Canavalia varieties, with UFA content dominating compared to saturated fatty acids. However, the lipid content in some fermented foods, such as chickpea tempeh, may decrease compared to unfermented flour (Reyes‐Moreno et al., 2004).

**TABLE 3:** Fatty acid percentage of the different formulations of Doowina during fermentation.

Day of fermentation		Fatty acids % in the different formulations of Doowina								
		P	P.T	P.Sq	P.S1	P.S1.T	P.S1.Sq	P.S2	P.S2.T	P.S2.Sq
Short‐chain fatty acids	0	5.06	6.11	6.93	5.46	6.52	7.38	5.8	6.97	7.75
	3	6.82	7.00	7.88	6.91	7.95	8.25	8.03	8.82	9.07
	6	6.74	7.23	8.23	7.49	8.31	8.72	8.15	8.96	9.28
	9	4.93	6.01	6.75	5.28	6.39	7.12	5.57	6.47	7.56
Saturated fatty acids	0	62.19	62.17	62.14	62.17	62.15	62.14	62.15	62.13	62.14
	3	61.07	60.93	60.87	60.64	60.5	60.44	59.95	59.81	59.75
	6	60.17	59.74	59.63	59.63	59.2	59.09	58.56	58.13	58.02
	9	59.81	59.02	58.95	59.13	58.34	58.27	57.92	57.13	57.06
Unsaturated fatty acids	0	30.11	30.48	31.07	31.25	31.74	32.37	32.7	33.38	33.79
	3	30.54	31.18	31.25	31.68	32.44	32.55	33.13	34.08	34.49
	6	30.95	31.31	31.37	32.09	32.57	32.67	33.54	34.21	34.62
	9	31.12	31.33	31.55	32.85	33.06	33.28	35.09	35.3	35.52

Abbreviations: P, primary mixture; P.S1, primary mixture + sourdough (0.5%); P.S1.Sq, primary mixture + sourdough (0.5%)  + butternut squash (8%); P.S1.T, primary mixture + sourdough (0.5%) + turnip (8%); P.S2, primary mixture + sourdough (1%); P.S2.Sq, primary mixture + sourdough (1%) + butternut squash (8%); P.S2.T, primary mixture + sourdough (1%) + turnip (8%); P.Sq, primary mixture + butternut squash (8%); P.T, primary mixture + turnip (8%).

### 3.6 Changes in the LAB community

**FIGURE 5:**
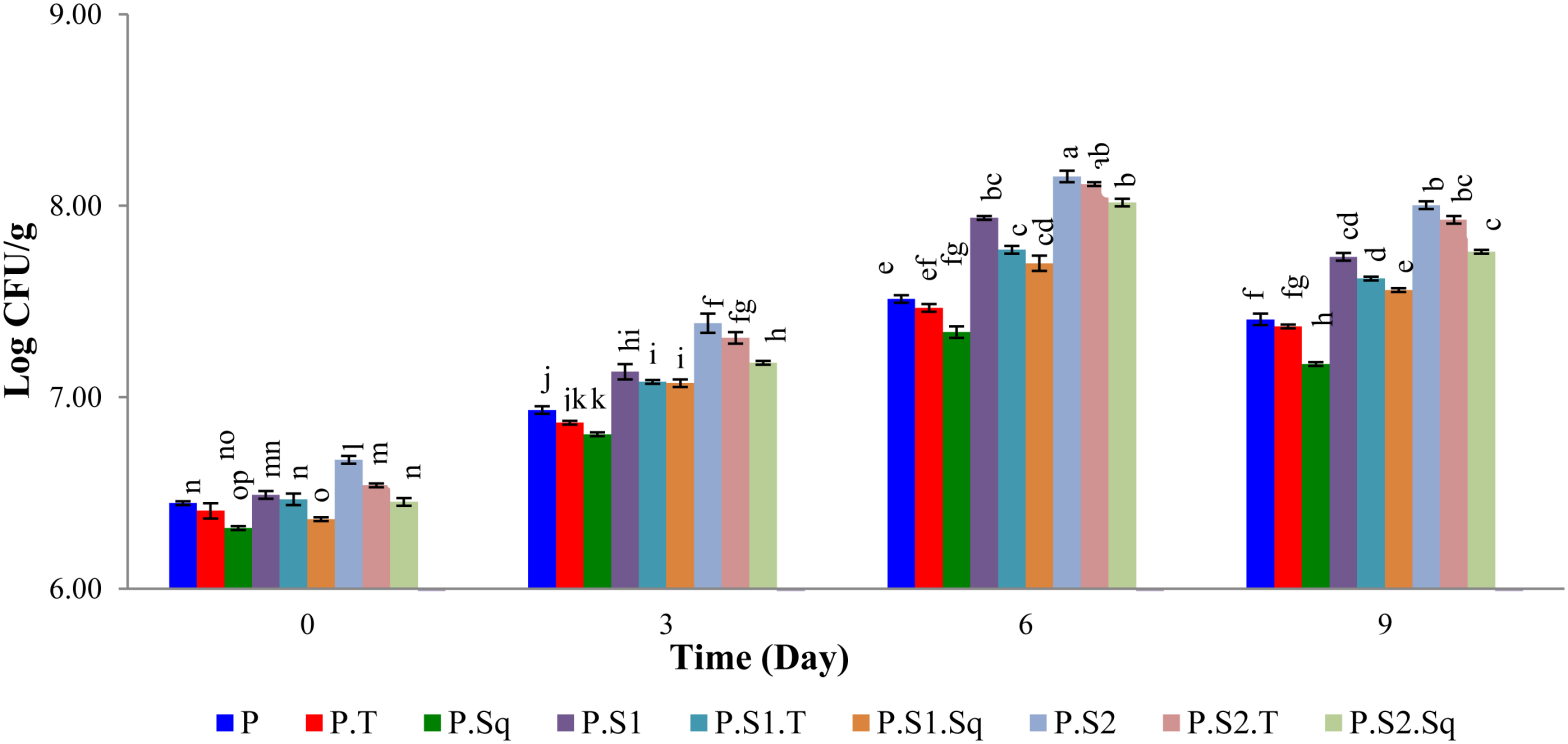
Effect of formulation and fermentation time on the LAB count in Doowina. Vertical bars represent the standard deviation (*n* = 3). Different letters indicate statistically significant differences (*p* < .05). P, primary mixture; P.T, primary mixture + turnip (8%); P.Sq, primary mixture + butternut squash (8%); P.S1, primary mixture + sourdough (0.5%); P.S1.T, primary mixture + sourdough (0.5%) + turnip (8%); P.S1.Sq, primary mixture + sourdough (0.5%) + butternut squash (8%); P.S2, primary mixture + sourdough (1%); P.S2.T, primary mixture + sourdough (1%) + turnip (8%); P.S2.Sq, primary mixture + sourdough (1%) + butternut squash (8%).

The number of LAB for samples containing sourdough, turnips, and butternut squash is shown in Figure 5. As shown in Figure 5, the number of LAB in the different formulations of Doowina was significantly different (*p* < .05). The LAB numbers increased until the 6th day of fermentation and then decreased until the 9th day of fermentation. Since sourdough contains LAB, the LAB community increased as the concentration of sourdough increased, so that in each fermentation period, the highest number of LAB belonged to the samples containing 1% sourdough and the lowest number belonged to the samples containing butternut squash. The results showed that the number of LAB decreased in the samples with turnips and butternut squash. This inhibitory effect on LAB growth could be due to the presence of antimicrobial compounds in these vegetables. The antimicrobial activity of turnip and butternut squash extracts was described by Ahmadvand and Sariri (2008) and Legaspi et al. (2011), respectively.

### 3.7 Sensory property analysis

**FIGURE 6:**
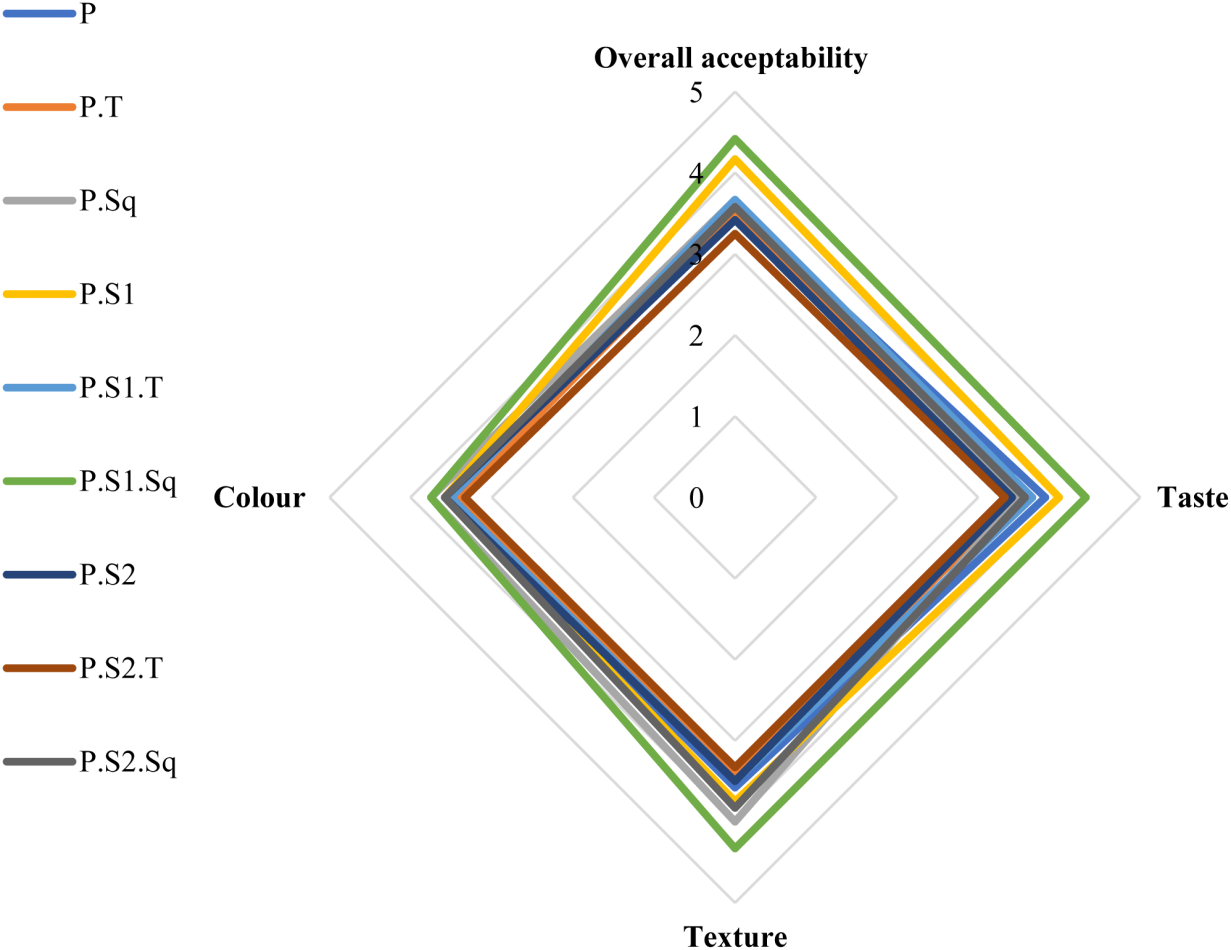
Consumer acceptability scores on a 5‐point scale for Doowina samples on the 9th day of fermentation. P, primary mixture; P.T, primary mixture + turnip (8%); P.Sq, Primary mixture + butternut squash (8%); P.S1, Primary mixture + sourdough (0.5%); P.S1.T, Primary mixture + sourdough (0.5%)  + turnip (8%); P.S1.Sq, Primary mixture + sourdough (0.5%) + butternut squash (8%); P.S2, Primary mixture + sourdough (1%); P.S2.T, Primary mixture + sourdough (1%) + turnip (8%); P.S2.Sq, Primary mixture + sourdough (1%) + butternut squash (8%).

The findings of the sensory properties of the samples fortified with sourdough, turnips, and butternut squash are shown in Figure 6. The results show that there were no significant differences (*p* < .05) in the sensory properties between the final products on the 9th day of fermentation.

The results showed that taste, color, and texture were acceptable to the panelists (score>3). The sensory properties of Doowina can be related to the chemical and enzymatic reactions that take place during fermentation and the interaction of microorganisms through different enzymatic activities (amylolytic, lipolytic, and proteolytic) (Mohammadi & Ostovar, 2023). The sensory properties of Doowina samples were affected by the turnip and the high concentration of sourdough, so their values decreased in the P.S2.T sample compared to the other samples. The lowest overall acceptability score was given to the P.S2.T sample (3.25), due to the presence of turnip and a high concentration (1%) of sourdough, resulting in an undesirable color and unpleasant taste due to the intense fermentation, while the highest overall acceptability score was given to the P.S1.Sq sample (4.42), which contained butternut squash and 0.5% sourdough, resulting in a desirable color and pleasant taste due to the mild fermentation. The microbial community in ayran and sourdough is probably responsible for improving the taste of the fermented product through the production of lactic acid, ethanol, carbon dioxide, and other microbial metabolites (Koca et al., 2002).

## 4 CONCLUSION

Nowadays, consumers intend to eat functional foods with superior properties. In this study, we formulated a new functional food based on Doowina with sourdough and vegetables. The fermentation process of the control sample, which did not contain sourdough, was carried out exclusively by the intrinsic microbial flora of the raw materials and the environment. The addition of sourdough, on the other hand, led to a more intense fermentation of Doowina due to its effective microbial flora. The microbial activity of the sourdough, especially in the presence of turnips and butternut squash, led to a greater release of phenolic compounds. As a result, the phenolic compound content and antioxidant activity of the samples containing sourdough, turnips, and butternut squash were higher than those of the control sample. The content of short‐chain fatty acids increased in the samples containing turnips, butternut squash, or sourdough. In addition, the highest content of linoleic acid was found in the samples containing butternut squash and sourdough. In terms of sensory properties, the samples with butternut squash and 0.5% sourdough achieved the highest values compared to the other samples. In conclusion, the samples of Doowina with butternut squash and sourdough as the best‐fermented samples showed more nutritional properties than other formulations of Doowina and can be introduced as a new functional food with health‐promoting properties. One of the limitations of this study is that the quality of the modified Doowina was only evaluated during fermentation. It is hoped that future studies will investigate the changes in quality characteristics and stability of the product during long storage to assess shelf life and consumer acceptance.

## AUTHOR CONTRIBUTIONS

**Sahar Bahrami:** Formal analysis (equal); investigation (equal); methodology (equal); software (equal); visualization (equal). **Nafiseh Davati:** Conceptualization (equal); data curation (equal); funding acquisition (equal); project administration (equal); resources (equal); supervision (equal); validation (equal); visualization (equal); writing – original draft (equal); writing – review and editing (equal). **Nooshin Noshirvani:** Data curation (equal); validation (equal); writing – review and editing (equal).

## FUNDING INFORMATION

This research did not receive any specific grants from funding agencies in the public, commercial, or not‐for‐profit sectors.

## CONFLICT OF INTEREST STATEMENT

The authors declare that they do not have any conflicts of interest.

## ETHICS STATEMENT

This study does not involve any human or animal testing.

## CONSENT TO PARTICIPATE

All the coauthors are willing to participate in this manuscript.

## CONSENT FOR PUBLICATION

All authors are willing to publish this manuscript.

## Supporting information


Data S1.

## Data Availability

Even though adequate data have been given in the form of tables and figures, all authors declare that if more data are required, then the data will be provided on a request basis.

## References

[fsn33915-bib-0001] Adebo, O. A. , & Gabriela Medina‐Meza, I. (2020). Impact of fermentation on the phenolic compounds and antioxidant activity of whole cereal grains: A mini review. Molecules, 25(4), 927. 10.3390/molecules25040927 32093014 PMC7070691

[fsn33915-bib-0002] Ahmadvand, S. , & Sariri, R. (2008). Antimicrobial activity of crude extracts of turnip (*Brassica rapa*). Journal of Pure and Applied Microbiology, 2(1), 193–196.

[fsn33915-bib-0003] Ahn‐Jarvis, J. H. , Riedl, K. M. , Schwartz, S. J. , & Vodovotz, Y. (2013). Design and selection of soy breads used for evaluating isoflavone bioavailability in clinical trials. Journal of Agricultural and Food Chemistry, 61(12), 3111–3120. 10.1021/jf304699k 23451757 PMC3804034

[fsn33915-bib-0004] AOAC . (2000). Official methods of analysis of AOAC (17th ed.). Association of Analytical Communities.

[fsn33915-bib-0005] Armesto, J. , Rocchetti, G. , Senizza, B. , Pateiro, M. , Barba, F. J. , Domínguez, R. , Lucini, L. , & Lorenzo, J. M. (2020). Nutritional characterization of butternut squash (*Cucurbita moschata D*.): Effect of variety (*Ariel vs. Pluto*) and farming type (conventional vs. organic). Food Research International, 132, 109052. 10.1016/j.foodres.2020.109052 32331650

[fsn33915-bib-0006] Auger, B. , Marnet, N. , Gautier, V. , Maia‐Grondard, A. , Leprince, F. , Renard, M. , Guyot, S. , Nesi, N. , & Routaboul, J.‐M. (2010). A detailed survey of seed coat flavonoids in developing seeds of *Brassica napus L* . Journal of Agricultural and Food Chemistry, 58(10), 6246–6256. 10.1021/jf903619v 20429588

[fsn33915-bib-0007] Balasubramanian, S. , Jincy, M. , Ramanathan, M. , Chandra, P. , & Deshpande, S. (2015). Studies on millet idli batter and its quality evaluation. International Food Research Journal, 22, 139–142.

[fsn33915-bib-0008] Bartkiene, E. , Juodeikiene, G. , Vidmantiene, D. , Viskelis, P. , & Urbonaviciene, D. (2011). Nutritional and quality aspects of wheat sourdough bread using *L. luteus* and *L. angustifolius* flours fermented by *Pedioccocus acidilactici* . International Journal of Food Science and Technology, 46(8), 1724–1733. 10.1111/j.1365-2621.2011.02668.x

[fsn33915-bib-0009] Bartolomé, B. , Estrella, I. , & Hernandez, T. (1997). Changes in phenolic compounds in lentils (*Lens culinaris*) during germination and fermentation. Zeitschrift für Lebensmitteluntersuchung Und‐Forschung A, 205(4), 290–294. 10.1007/s002170050167

[fsn33915-bib-0010] Bilgiçli, N. , Elgün, A. , Herken, E. N. , Ertaş, N. , & İbanogˇlu, Ş. (2006). Effect of wheat germ/bran addition on the chemical, nutritional and sensory quality of Tarhana, a fermented wheat flour‐yoghurt product. Journal of Food Engineering, 77(3), 680–686. 10.1016/j.jfoodeng.2005.07.030

[fsn33915-bib-0011] Bilgiçli, N. , Elgün, A. , & Türker, S. (2006). Effects of various phytase sources on phytic acid content, mineral extractability and protein digestibility of Tarhana. Food Chemistry, 98(2), 329–337. 10.1016/j.foodchem.2005.05.078

[fsn33915-bib-0012] Bligh, E. G. , & Dyer, W. J. (1959). A rapid method of total lipid extraction and purification. Canadian Journal of Biochemistry and Physiology, 37(8), 911–917. 10.1139/o59-099 13671378

[fsn33915-bib-0013] Bochkov, D. V. , Sysolyatin, S. V. , Kalashnikov, A. I. , & Surmacheva, I. A. (2012). Shikimic acid: Review of its analytical, isolation, and purification techniques from plant and microbial sources. Journal of Chemical Biology, 5(1), 5–17. 10.1007/s12154-011-0064-8 22826715 PMC3251648

[fsn33915-bib-0014] Bradford, M. M. (1976). A rapid and sensitive method for the quantitation of microgram quantities of protein utilizing the principle of protein‐dye binding. Analytical Biochemistry, 72(1–2), 248–254. 10.1016/0003-2697(76)90527-3 942051

[fsn33915-bib-0015] Bradley, D. (2005). Star role for bacteria in controlling flu pandemic? Nature Reviews Drug Discovery, 4(12), 945–947. 10.1038/nrd1917 16370070

[fsn33915-bib-0016] Brand‐Williams, W. , Cuvelier, M.‐E. , & Berset, C. (1995). Use of a free radical method to evaluate antioxidant activity. LWT‐ Food Science and Technology, 28(1), 25–30. 10.1016/s0023-6438(95)80008-5

[fsn33915-bib-0017] Cao, Q. , Wang, G. , & Peng, Y. (2021). A critical review on phytochemical profile and biological effects of turnip (*Brassica rapa L*.). Frontiers in Nutrition, 8, 721733. 10.3389/fnut.2021.721733 34395503 PMC8360391

[fsn33915-bib-0018] Çelik, I. , Işik, F. , Şimşek, Ö. , & Gürsoy, O. (2005). The effects of the addition of baker's yeast on the functional properties and quality of Tarhana, a traditional fermented food. Czech Journal of Food Sciences, 23, 190–195. 10.17221/3390-cjfs

[fsn33915-bib-0019] Chang, H.‐H. , Chen, C.‐S. , & Lin, J.‐Y. (2009). Dietary perilla oil lowers serum lipids and ovalbumin‐specific IgG1, but increases total IgE levels in ovalbumin‐challenged mice. Food and Chemical Toxicology, 47(4), 848–854. 10.1016/j.fct.2009.01.017 19271319

[fsn33915-bib-0020] Coda, R. , Di Cagno, R. , Gobbetti, M. , & Rizzello, C. G. (2014). Sourdough lactic acid bacteria: Exploration of non‐wheat cereal‐based fermentation. Food Microbiology, 37, 51–58. 10.1016/j.fm.2013.06.018 24230473

[fsn33915-bib-0021] Coda, R. , Di Cagno, R. , Rizzello, C. G. , Nionelli, L. , Edema, M. O. , & Gobbetti, M. (2011). Utilization of African grains for sourdough bread making. Journal of Food Science, 76(6), M329–M335. 10.1111/j.1750-3841.2011.02240.x 22417505

[fsn33915-bib-0022] Coda, R. , Melama, L. , Rizzello, C. G. , Curiel, J. A. , Sibakov, J. , Holopainen, U. , Pulkkinen, M. , & Sozer, N. (2015). Effect of air classification and fermentation by *Lactobacillus plantarum* VTT E‐133328 on faba bean (*Vicia faba L*.) flour nutritional properties. International Journal of Food Microbiology, 193, 34–42. 10.1016/j.ijfoodmicro.2014.10.012 25462921

[fsn33915-bib-0023] Coda, R. , Rizzello, C. G. , & Gobbetti, M. (2010). Use of sourdough fermentation and pseudo‐cereals and leguminous flours for the making of a functional bread enriched of γ‐aminobutyric acid (GABA). International Journal of Food Microbiology, 137(2–3), 236–245. 10.1016/j.ijfoodmicro.2009.12.010 20071045

[fsn33915-bib-0024] Curiel, J. A. , Coda, R. , Centomani, I. , Summo, C. , Gobbetti, M. , & Rizzello, C. G. (2015). Exploitation of the nutritional and functional characteristics of traditional Italian legumes: The potential of sourdough fermentation. International Journal of Food Microbiology, 196, 51–61. 10.1016/j.ijfoodmicro.2014.11.032 25522057

[fsn33915-bib-0025] Đorđević, T. M. , Šiler‐Marinković, S. S. , & Dimitrijević‐Branković, S. I. (2010). Effect of fermentation on antioxidant properties of some cereals and pseudo cereals. Food Chemistry, 119(3), 957–963. 10.1016/j.foodchem.2009.07.049

[fsn33915-bib-0026] Dueñas, M. , Fernández, D. , Hernández, T. , Estrella, I. , & Muñoz, R. (2005). Bioactive phenolic compounds of cowpeas (*Vigna sinensis L*). Modifications by fermentation with natural microflora and with *Lactobacillus plantarum* ATCC 14917. Journal of the Science of Food and Agriculture, 85(2), 297–304. 10.1002/jsfa.1924

[fsn33915-bib-0027] Erbaş, M. , Certel, M. , & Uslu, M. K. (2005). Microbiological and chemical properties of Tarhana during fermentation and storage as wet—Sensorial properties of Tarhana soup. LWT‐ Food Science and Technology, 38(4), 409–416. 10.1016/j.lwt.2004.06.009

[fsn33915-bib-0028] Erbaş, M. , Uslu, M. K. , Erbaş, M. O. , & Certel, M. (2006). Effects of fermentation and storage on the organic and fatty acid contents of Tarhana, a Turkish fermented cereal food. Journal of Food Composition and Analysis, 19(4), 294–301. 10.1016/j.jfca.2004.12.002

[fsn33915-bib-0029] Erkan, H. , Çelik, S. , Bilgi, B. , & Köksel, H. (2006). A new approach for the utilization of barley in food products: Barley Tarhana. Food Chemistry, 97(1), 12–18. 10.1016/j.foodchem.2005.03.018

[fsn33915-bib-0030] Esteban‐Torres, M. , Landete, J. M. , Reverón, I. , Santamaría, L. , de las Rivas, B. , & Muñoz, R. (2015). A *Lactobacillus plantarum* esterase active on a broad range of phenolic esters. Applied and Environmental Microbiology, 81(9), 3235–3242. 10.1128/aem.00323-15 25746986 PMC4393438

[fsn33915-bib-0031] Ferreira, L. R. , Macedo, J. A. , Ribeiro, M. L. , & Macedo, G. A. (2013). Improving the chemopreventive potential of orange juice by enzymatic biotransformation. Food Research International, 51(2), 526–535. 10.1016/j.foodres.2013.01.018

[fsn33915-bib-0032] Francisco, M. , Moreno, D. A. , Cartea, M. E. , Ferreres, F. , García‐Viguera, C. , & Velasco, P. (2009). Simultaneous identification of glucosinolates and phenolic compounds in a representative collection of vegetable *Brassica rapa* . Journal of Chromatography A, 1216(38), 6611–6619. 10.1016/j.chroma.2009.07.055 19683241

[fsn33915-bib-0033] Frias, J. , Martinez‐Villaluenga, C. , & Peñas, E. (2016). Fermented Foods in Health and Disease Prevention. Academic Press. 10.1016/b978-0-12-802309-9.00002-9

[fsn33915-bib-0034] Gabrial, S. , Zaghloul, A. H. , Khalaf‐Allah, A. , El‐Shimi, N. M. , Mohamed, R. S. , & Gabrial, G. N. (2010). Synbiotic Tarhana as a functional food. Journal of American Science, 6(12), 1402–1412.

[fsn33915-bib-0035] Gabriele, M. , Arouna, N. , Árvay, J. , Longo, V. , & Pucci, L. (2023). Sourdough fermentation improves the antioxidant, antihypertensive, and anti‐inflammatory properties of *Triticum dicoccum* . International Journal of Molecular Sciences, 24(7), 6283. 10.3390/ijms24076283 37047259 PMC10094579

[fsn33915-bib-0036] Gabriele, M. , Sparvoli, F. , Bollini, R. , Lubrano, V. , Longo, V. , & Pucci, L. (2019). The impact of sourdough fermentation on non‐nutritive compounds and antioxidant activities of flours from different *Phaseolus vulgaris* L. genotypes. Journal of Food Science, 84(7), 1929–1936. 10.1111/1750-3841.14672 31218698

[fsn33915-bib-0038] Ghafoori, F. , & Hosseini Ghaboos, H. (2018). Physicochemical and sensory characteristics of breakfast cereals blended with pumpkin powder. Iranian Journal of Nutrition Sciences and Food Technology, 13(1), 95–104.

[fsn33915-bib-0039] Gocmen, D. , Gurbuz, O. , Rouseff, R. L. , Smoot, J. M. , & Fatih Dagdelen, A. (2004). Gas chromatographic‐olfactometric characterization of aroma active compounds in sun‐dried and vacuum‐dried Tarhana. European Food Research and Technology, 218(6), 573–578. 10.1007/s00217-004-0913-6

[fsn33915-bib-0040] Gurbuz, O. , Gocmen, D. , Ozmen, N. , & Dagdelen, F. (2010). Effects of yeast, fermentation time, and preservation methods on Tarhana. Preparative Biochemistry and Biotechnology, 40(4), 263–275. 10.1080/10826068.2010.488987 21108130

[fsn33915-bib-0041] Haș, I. M. , Vodnar, D.‐C. , Bungau, A. F. , Tarce, A. G. , Tit, D. M. , & Teleky, B.‐E. (2023). Enhanced elderberry snack bars: A sensory, nutritional, and rheological evaluation. Food, 12(19), 3544. 10.3390/foods12193544 PMC10572914 37835197

[fsn33915-bib-0042] Hassan, M. F. , & Gadallah, M. G. (2018). Physico‐chemical and sensory properties of Tarhana prepared from different cereals and dairy ingredients. Current Journal of Applied Science and Technology, 29(3), 1–14. 10.9734/cjast/2018/38459

[fsn33915-bib-0044] Iso, P. (2000). Animal and vegetable fats and oils–preparation of methyl esters of fatty acids. Polish Standard Method PN‐EN ISO, 5509, 2000. 10.3403/02020190u

[fsn33915-bib-0045] Javed, A. , Ahmad, A. , Nouman, M. , Hameed, A. , Tahir, A. , & Shabbir, U. (2019). Turnip (*Brassica Rapus L*.): A natural health tonic. Brazilian Journal of Food Technology, 22, e2018253. 10.1590/1981-6723.25318

[fsn33915-bib-0046] Jiménez, N. , Esteban‐Torres, M. , Mancheño, J. M. , de Las Rivas, B. , & Muñoz, R. (2014). Tannin degradation by a novel tannase enzyme present in some *Lactobacillus plantarum* strains. Applied and Environmental Microbiology, 80(10), 2991–2997. 10.1128/aem.00324-14 24610854 PMC4018929

[fsn33915-bib-0047] Katina, K. , Laitila, A. , Juvonen, R. , Liukkonen, K.‐H. , Kariluoto, S. , Piironen, V. , Landberg, R. , Åman, P. , & Poutanen, K. (2007). Bran fermentation as a means to enhance technological properties and bioactivity of rye. Food Microbiology, 24(2), 175–186. 10.1016/j.fm.2006.07.012 17008162

[fsn33915-bib-0048] Khattab, R. , & Arntfield, S. (2009). Nutritional quality of legume seeds as affected by some physical treatments 2. Antinutritional factors. LWT‐ Food Science and Technology, 42(6), 1113–1118. 10.1016/j.lwt.2009.02.004

[fsn33915-bib-0049] Khattab, R. , Arntfield, S. , & Nyachoti, C. (2009). Nutritional quality of legume seeds as affected by some physical treatments, part 1: Protein quality evaluation. LWT‐ Food Science and Technology, 42(6), 1107–1112. 10.1016/j.lwt.2009.02.008

[fsn33915-bib-0050] Kivanc, M. , & Funda, E. G. (2017). A functional food: A traditional Tarhana fermentation. Food Science and Technology, 37, 269–274. 10.1590/1678-457x.08815

[fsn33915-bib-0051] Koca, A. , Yazici, F. , & Anil, M. (2002). Utilization of soy yoghurt in Tarhana production. European Food Research and Technology, 215(4), 293–297. 10.1007/s00217-002-0568-0

[fsn33915-bib-0052] Legaspi, M. A. G. , Tiongco, A. F. Y. , & Faith, A. (2011). Screening for the antimicrobial properties of vegetable extracts on selected test microorganisms.

[fsn33915-bib-0053] Leroy, F. , & De Vuyst, L. (2004). Lactic acid bacteria as functional starter cultures for the food fermentation industry. Trends in Food Science and Technology, 15(2), 67–78. 10.1016/j.tifs.2003.09.004

[fsn33915-bib-0054] Limón, R. I. , Peñas, E. , Torino, M. I. , Martínez‐Villaluenga, C. , Dueñas, M. , & Frias, J. (2015). Fermentation enhances the content of bioactive compounds in kidney bean extracts. Food Chemistry, 172, 343–352. 10.1016/j.foodchem.2014.09.084 25442563

[fsn33915-bib-0055] María Landete, J. , Hernández, T. , Robredo, S. , Duenas, M. , de Las Rivas, B. , Estrella, I. , & Munoz, R. (2015). Effect of soaking and fermentation on content of phenolic compounds of soybean (*Glycine max* cv. Merit) and mung beans (*Vigna radiata* [L] Wilczek). International Journal of Food Sciences and Nutrition, 66(2), 203–209. 10.3109/09637486.2014.986068 25582183

[fsn33915-bib-0056] Martins, S. , Mussatto, S. I. , Martínez‐Avila, G. , Montañez‐Saenz, J. , Aguilar, C. N. , & Teixeira, J. A. (2011). Bioactive phenolic compounds: Production and extraction by solid‐state fermentation. A review. Biotechnology Advances, 29(3), 365–373. 10.1016/j.biotechadv.2011.01.008 21291993

[fsn33915-bib-0057] Mashak, Z. , Sodagari, H. , Mashak, B. , & Niknafs, S. (2014). Chemical and microbial properties of two Iranian traditional fermented cereal‐dairy based foods: Kashk‐e Zard and Tarkhineh. International Journal of Biosciences, 4(12), 124–133. 10.12692/ijb/4.12.124-133

[fsn33915-bib-0058] Michalski, M. C. (2009). Specific molecular and colloidal structures of milk fat affecting lipolysis, absorption and postprandial lipemia. European Journal of Lipid Science and Technology, 111(5), 413–431. 10.1002/ejlt.200800254

[fsn33915-bib-0059] Mohammadi, N. , & Ostovar, N. (2023). Chemical composition, fatty acid composition, and volatile compounds of a traditional Kurdish fermented cereal food: Tarkhineh. Food Chemistry Advances, 2, 100187. 10.1016/j.focha.2023.100187

[fsn33915-bib-0060] Moradi, L. , Paimard, G. , Sadeghi, E. , Rouhi, M. , Mohammadi, R. , Noroozi, R. , & Safajoo, S. (2022). Fate of aflatoxins M1 and B1 within the period of production and storage of Tarkhineh: A traditional Persian fermented food. Food Science & Nutrition, 10(3), 945–952. 10.1002/fsn3.2728 35311167 PMC8907732

[fsn33915-bib-0061] Moroni, A. V. , Zannini, E. , Sensidoni, G. , & Arendt, E. K. (2012). Exploitation of buckwheat sourdough for the production of wheat bread. European Food Research and Technology, 235(4), 659–668. 10.1007/s00217-012-1790-z

[fsn33915-bib-0062] Morton, L. W. , Caccetta, R. A. A. , Puddey, I. B. , & Croft, K. D. (2000). Chemistry and biological effects of dietary phenolic compounds: Relevance to cardiovascular disease. Clinical and Experimental Pharmacology and Physiology, 27(3), 152–159. 10.1046/j.1440-1681.2000.03214.x 10744340

[fsn33915-bib-0063] Newmark, H. L. (1996). Plant phenolics as potential cancer prevention agents. Advances in Experimental Medicine and Biology, 401, 25–34. 10.1007/978-1-4613-0399-2_3 8886124

[fsn33915-bib-0064] Niveditha, V. , Sridhar, K. , & Chatra, S. (2012). Fatty acid composition of cooked and fermented beans of the wild legumes (Canavalia) of coastal sand dunes. International Food Research Journal, 19(4), 1401–1407. 10.1016/b978-0-12-811412-4.00008-4

[fsn33915-bib-0065] O'Callaghan, Y. C. , Shevade, A. V. , Guinee, T. P. , O'Connor, T. P. , & O'Brien, N. M. (2019). Comparison of the nutritional composition of experimental fermented milk: Wheat bulgur blends and commercially available Kishk and Tarhana products. Food Chemistry, 278, 110–118. 10.1016/j.foodchem.2018.11.026 30583351

[fsn33915-bib-0066] Omedi, J. O. , Huang, W. , & Zheng, J. (2019). Effect of sourdough lactic acid bacteria fermentation on phenolic acid release and antifungal activity in pitaya fruit substrate. LWT, 111, 309–317. 10.1016/j.lwt.2019.05.038

[fsn33915-bib-0067] Othman, N. B. , Roblain, D. , Chammen, N. , Thonart, P. , & Hamdi, M. (2009). Antioxidant phenolic compounds loss during the fermentation of Chétoui olives. Food Chemistry, 116(3), 662–669. 10.1016/j.foodchem.2009.02.084

[fsn33915-bib-0068] Ovando‐Martinez, M. , Daglioglu, O. , Gecgel, U. , & Simsek, S. (2014). Analysis of the fatty acids and phenolic compounds in a cereal‐based fermented food (Tarhana). Food and Nutrition Sciences, 2014, 1177–1184. 10.4236/fns.2014.513128

[fsn33915-bib-0069] Ozdemir, S. , Gocmen, D. , & Yildirim Kumral, A. (2007). A traditional Turkish fermented cereal food: Tarhana. Food Reviews International, 23(2), 107–121. 10.1080/87559120701224923

[fsn33915-bib-0070] Palanisamy, B. D. , Rajendran, V. , Sathyaseelan, S. , Bhat, R. , & Venkatesan, B. P. (2012). Enhancement of nutritional value of finger millet‐based food (Indian dosa) by co‐fermentation with horse gram flour. International Journal of Food Sciences and Nutrition, 63(1), 5–15. 10.3109/09637486.2011.591367 21696301

[fsn33915-bib-0071] Peñaloza‐Espinosa, J. , Gloria, J. , Mora‐Escobedo, R. , Chanona‐Pérez, J. , Farrera‐Rebollo, R. , & Calderón‐Domínguez, G. (2011). Sourdough and bread properties as affected by soybean protein addition. In Soybean‐Applications and Technology. IntechOpen. 10.5772/15567

[fsn33915-bib-0072] Pommerville, J. C. (2007). Alcamo's laboratory fundamentals of microbiology. Jones & Bartlett Learning.

[fsn33915-bib-0073] Prinyawiwatkul, W. , Beuchat, L. , McWatters, K. , & Phillips, R. (1996). Changes in fatty acid, simple sugar, and oligosaccharide content of cowpea (*Vigna unguiculata*) flour as a result of soaking, boiling, and fermentation with *Rhizopus microsporus* var. *oligosporus* . Food Chemistry, 57(3), 405–413. 10.1016/0308-8146(95)00242-1

[fsn33915-bib-0074] Reyes‐Moreno, C. , Cuevas‐Rodríguez, E. , Milán‐Carrillo, J. , Cárdenas‐Valenzuela, O. , & Barrón‐Hoyos, J. (2004). Solid state fermentation process for producing chickpea (*Cicer arietinum L*) tempeh flour. Physicochemical and nutritional characteristics of the product. Journal of the Science of Food and Agriculture, 84(3), 271–278. 10.1002/jsfa.1637

[fsn33915-bib-0075] Rizzello, C. G. , Calasso, M. , Campanella, D. , De Angelis, M. , & Gobbetti, M. (2014). Use of sourdough fermentation and mixture of wheat, chickpea, lentil and bean flours for enhancing the nutritional, texture and sensory characteristics of white bread. International Journal of Food Microbiology, 180, 78–87. 10.1016/j.ijfoodmicro.2014.04.005 24794619

[fsn33915-bib-0076] Rodríguez, H. , Curiel, J. A. , Landete, J. M. , de las Rivas, B. , de Felipe, F. L. , Gómez‐Cordovés, C. , Mancheño, J. M. , & Muñoz, R. (2009). Food phenolics and lactic acid bacteria. International Journal of Food Microbiology, 132(2–3), 79–90. 19419788 10.1016/j.ijfoodmicro.2009.03.025

[fsn33915-bib-0077] Scalzo, R. L. , Genna, A. , Branca, F. , Chedin, M. , & Chassaigne, H. (2008). Anthocyanin composition of cauliflower (*Brassica oleracea L*. var. *botrytis*) and cabbage (*B. Oleracea L*. var. *capitata*) and its stability in relation to thermal treatments. Food Chemistry, 107(1), 136–144. 10.1016/j.foodchem.2007.07.072

[fsn33915-bib-0078] Sharma, G. , Srivastava, A. K. , & Prakash, D. (2011). Phytochemicals of nutraceutical importance: Their role in health and diseases. Pharmacology, 2, 408–427.

[fsn33915-bib-0079] Singleton, V. L. , Orthofer, R. , & Lamuela‐Raventós, R. M. (1999). Analysis of total phenols and other oxidation substrates and antioxidants by means of folin‐ciocalteu reagent. In Methods in enzymology (Vol. 299, pp. 152–178). Elsevier. 10.1016/s0076-6879(99)99017-1

[fsn33915-bib-0080] Soni, S. , Sandhu, D. , & Vilkhu, K. (1985). Studies on dosa—An indigenous Indian fermented food: Some biochemical changes accompanying fermentation. Food Microbiology, 2(3), 175–181. 10.1016/0740-0020(85)90032-2

[fsn33915-bib-0081] Soyuçok, A. , Yurt, M. N. Z. , Altunbas, O. , Ozalp, V. C. , & Sudagidan, M. (2021). Metagenomic and chemical analysis of Tarhana during traditional fermentation process. Food Bioscience, 39, 100824. 10.1016/j.fbio.2020.100824

[fsn33915-bib-0082] Steinkraus, K. (2018). Handbook of indigenous fermented foods, revised and expanded. CRC press. 10.1201/9780203752821

[fsn33915-bib-0083] Sumarmono, J. , & Sulistyowati, M. (2015). Fatty acids profiles of fresh milk, yogurt and concentrated yogurt from peranakan Etawah goat milk. Procedia Food Science, 3, 216–222. 10.1016/j.profoo.2015.01.024

[fsn33915-bib-0084] Tarakci, Z. , Anil, M. , Koca, I. , & Islam, A. (2013). Effects of adding cherry laurel (*Laurocerasus officinalis*) on some physicochemical and functional properties and sensorial quality of Tarhana. Quality Assurance & Safety of Crops and Food, 5(4), 347–355. 10.3920/qas2012.0155

[fsn33915-bib-0085] Teleky, B.‐E. , Martău, G. A. , Ranga, F. , Pop, I. D. , & Vodnar, D. C. (2022). Biofunctional soy‐based sourdough for improved rheological properties during storage. Scientific Reports, 12(1), 17535. 10.1038/s41598-022-22551-z 36266426 PMC9584935

[fsn33915-bib-0086] Torino, M. I. , Limón, R. I. , Martínez‐Villaluenga, C. , Mäkinen, S. , Pihlanto, A. , Vidal‐Valverde, C. , & Frias, J. (2013). Antioxidant and antihypertensive properties of liquid and solid state fermented lentils. Food Chemistry, 136(2), 1030–1037. 10.1016/j.foodchem.2012.09.015 23122159

[fsn33915-bib-0087] Xiong, T. , Guan, Q. , Song, S. , Hao, M. , & Xie, M. (2012). Dynamic changes of lactic acid bacteria flora during Chinese sauerkraut fermentation. Food Control, 26(1), 178–181. 10.1016/j.foodcont.2012.01.027

[fsn33915-bib-0088] Zapaśnik, A. , Sokołowska, B. , & Bryła, M. (2022). Role of lactic acid bacteria in food preservation and safety. Food, 11(9), 1283. 10.3390/foods11091283 PMC9099756 35564005

[fsn33915-bib-0089] Zomorod, S. , Heidari, R. , & Behnam, S. (2020). The effect of pumpkin powder on the quality properties of gluten‐free sponge cake prepared with corn flour. Food Engineering Research, 18(67), 1–14.

