# Supplementary material for: The effect of sourdough, turnips, and butternut squash on the physicochemical and nutritional properties of Doowina functional food during fermentation
Source: Food Sci Nutr. 2024 Jan 2;12(3):2131–44. doi: 10.1002/fsn3.3915 (PMC10916581; doi:10.1002/fsn3.3915)
Supplement: Supplementary file 1 — Data S1. [file FSN3-12-2131-s001.docx]

**Supplementary file 1** Fatty acids% profile of the different formulations of Doowina during fermentation

|  | **Days** | **P** | **P. T** | **P. Sq** | **P. S1** | **P. S1.T** | **P. S1.Sq** | **P. S2** | **P. S2.T** | **P. S2.Sq** |
| --- | --- | --- | --- | --- | --- | --- | --- | --- | --- | --- |
| Butryic- C4 | 0 | 2.23 | 0.21 | 0.07 | 1.98 | 1.35 | 1.70 | 1.53 | 2.03 | 0.74 |
|  | 3 | 2.214 | 1.67166 | 0.2494 | 3.0688 | 0.9622 | 1.6962 | 1.9881 | 1.1834 | 0.74 |
|  | 6 | 2.302 | 1.9778 | 1.053 | 1.441 | 0.912 | 1.599 | 1.512 | 1.8755 | 1.888 |
|  | 9 | 0.91 | 1.69 | 1.92 | 1.68 | 1.44 | 0.23 | 1.30 | 1.88 | 1.19 |
| Caproic- C6 | 0 | 1.77 | 0.41 | 0.19 | 1.61 | 1.20 | 1.39 | 1.35 | 1.64 | 1.21 |
|  | 3 | 1.8144 | 1.4907 | 0.5089 | 2.1562 | 1.0907 | 1.4832 | 1.2278 | 1.2598 | 1.2092 |
|  | 6 | 1.794 | 1.744 | 1.075 | 1.276 | 0.938 | 1.347 | 1.192 | 1.4001 | 0.9586 |
|  | 9 | 0.89 | 1.23 | 1.51 | 1.15 | 1.11 | 0.45 | 1.12 | 1.40 | 0.96 |
| Caprylic- C8 | 0 | 1.15 | 0.52 | 0.33 | 1.11 | 0.88 | 0.96 | 0.98 | 0.99 | 0.81 |
|  | 3 | 1.2509 | 1.1213 | 0.5922 | 1.3127 | 0.8813 | 1.602 | 0.7783 | 0.9514 | 0.8072 |
|  | 6 | 1.2 | 1.086 | 0.882 | 0.965 | 0.756 | 0.944 | 0.929 | 0.8806 | 0.6824 |
|  | 9 | 0.75 | 0.80 | 1.02 | 0.76 | 0.73 | 0.51 | 0.84 | 0.88 | 0.68 |
| Capric- C10 | 0 | 2.60 | 1.79 | 1.30 | 2.68 | 2.28 | 2.36 | 2.47 | 2.23 | 2.15 |
|  | 3 | 2.9672 | 2.7187 | 2.065 | 2.7299 | 2.3493 | 2.4576 | 1.6391 | 2.388 | 2.1489 |
|  | 6 | 2.991 | 2.578 | 2.339 | 2.366 | 2.062 | 2.115 | 2.218 | 2.0875 | 1.566 |
|  | 9 | 1.99 | 1.90 | 2.38 | 1.69 | 1.86 | 1.73 | 2.07 | 2.09 | 1.57 |
| Lauric- C12 | 0 | 3.08 | 2.66 | 2.21 | 3.27 | 2.86 | 2.92 | 3.04 | 2.68 | 2.93 |
|  | 3 | 3.3342 | 3.1737 | 2.9535 | 2.9139 | 2.9741 | 2.8355 | 2.0429 | 2.9433 | 2.9311 |
|  | 6 | 3.258 | 3.017 | 2.906 | 3.081 | 2.764 | 2.668 | 2.485 | 2.628 | 2.0278 |
|  | 9 | 2.61 | 2.29 | 2.72 | 2.01 | 2.28 | 2.54 | 2.45 | 2.63 | 2.03 |
| Myristic- C14 | 0 | 9.70 | 9.48 | 8.53 | 10.21 | 9.34 | 9.42 | 9.58 | 8.12 | 9.71 |
|  | 3 | 9.7054 | 9.496 | 10.0527 | 8.7625 | 9.1109 | 8.8072 | 6.533 | 9.4364 | 9.7081 |
|  | 6 | 9.767 | 9.292 | 9.179 | 8.981 | 8.937 | 8.765 | 8.31 | 7.6468 | 6.551 |
|  | 9 | 8.46 | 7.66 | 8.45 | 6.43 | 7.25 | 8.29 | 7.79 | 7.65 | 6.55 |
| Myristoleic- C14-1 | 0 | 0.93 | 0.83 | 0.73 | 0.99 | 0.88 | 0.89 | 0.89 | 0.84 | 0.93 |
|  | 3 | 0.9137 | 1.0033 | 0.8893 | 0.9544 | 0.8758 | 0.8776 | 0.6975 | 0.9732 | 0.9338 |
|  | 6 | 0.902 | 0.886 | 0.855 | 0.87 | 0.839 | 0.809 | 0.744 | 0.744 | 0.5916 |
|  | 9 | 0.79 | 0.72 | 0.82 | 0.62 | 0.70 | 0.79 | 0.72 | 0.74 | 0.59 |
| Pentadecanoic-C15 | 0 | 0.61 | 0.52 | 0.51 | 0.00 | 0.52 | 0.51 | 0.53 | 0.47 | 0.58 |
|  | 3 | 0.5114 | 0.485 | 0.5089 | 0.5725 | 0.4747 | 0.4925 | 0.4222 | 0.5244 | 0.5754 |
|  | 6 | 0.521 | 0.496 | 0.49 | 0.527 | 0.518 | 0.475 | 0.462 | 0.4296 | 0.141 |
|  | 9 | 0.46 | 0.41 | 0.47 | 0.37 | 0.39 | 0.50 | 0.42 | 0.43 | 0.14 |
| cis-10-Pentadecannoic - C15-1 | 0 | 1.15 | 1.29 | 1.16 | 1.27 | 1.18 | 1.16 | 1.18 | 0.99 | 1.24 |
|  | 3 | 1.771 | 1.1911 | 1.2967 | 1.1384 | 1.1927 | 1.0908 | 0.888 | 1.1472 | 1.2379 |
|  | 6 | 1.198 | 1.64 | 1.176 | 1.22 | 1.196 | 1.077 | 1.078 | 0.9521 | 0.8407 |
|  | 9 | 1.08 | 0.97 | 1.07 | 0.88 | 0.91 | 1.10 | 0.98 | 0.95 | 0.84 |
| Palmitic - C16 | 0 | 29.82 | 33.35 | 33.25 | 32.22 | 32.01 | 31.77 | 32.00 | 29.02 | 31.51 |
|  | 3 | 30.7758 | 30.2017 | 33.5627 | 29.7301 | 31.5861 | 30.4426 | 28.7357 | 29.6203 | 31.5143 |
|  | 6 | 30.703 | 31.102 | 31.384 | 31.182 | 32.315 | 31.062 | 30.568 | 31.3606 | 30.2219 |
|  | 9 | 30.91 | 29.30 | 29.66 | 29.23 | 29.08 | 31.82 | 29.90 | 31.36 | 30.22 |
| Palmitoleic - C16-1 | 0 | 1.65 | 1.76 | 1.73 | 1.84 | 1.83 | 1.77 | 1.67 | 1.95 | 1.51 |
|  | 3 | 1.7005 | 1.8563 | 1.8725 | 1.7371 | 1.8592 | 1.8886 | 2.1394 | 2.0602 | 1.5121 |
|  | 6 | 1.629 | 1.64 | 1.621 | 1.729 | 1.715 | 1.621 | 1.63 | 1.8886 | 0.6603 |
|  | 9 | 1.57 | 1.48 | 1.63 | 1.34 | 1.46 | 1.70 | 1.54 | 1.89 | 0.66 |
| Heptadecanoic- C17 | 0 | 0.57 | 0.60 | 0.59 | 0.60 | 0.62 | 0.53 | 0.60 | 0.47 | 1.35 |
|  | 3 | 0.5558 | 0.584 | 0.6267 | 0.4475 | 0.5427 | 0.572 | 0.4671 | 0.5153 | 1.35 |
|  | 6 | 0.576 | 0.563 | 0.615 | 0.571 | 0.588 | 0.519 | 0.498 | 0.4055 | 1.6228 |
|  | 9 | 0.55 | 0.49 | 0.60 | 0.44 | 0.43 | 0.56 | 0.49 | 0.41 | 1.62 |
| cis-10-Heptadecenoic- C17-1 | 0 | 0.55 | 0.66 | 0.55 | 0.49 | 0.53 | 0.50 | 0.50 | 0.45 | 0.83 |
|  | 3 | 0.5019 | 0.5289 | 0.475 | 0.3611 | 0.4647 | 0.4945 | 0.3564 | 0.4705 | 0.8308 |
|  | 6 | 0.495 | 0.517 | 0.501 | 0.506 | 0.532 | 0.489 | 0.611 | 0.3484 | 0.5121 |
|  | 9 | 0.49 | 0.43 | 0.47 | 0.38 | 0.41 | 0.45 | 0.45 | 0.35 | 0.51 |
| Stearic - C18-0 | 0 | 7.26 | 6.50 | 7.17 | 6.14 | 6.40 | 6.04 | 6.09 | 5.85 | 6.67 |
|  | 3 | 9.0094 | 5.4596 | 5.1791 | 4.8762 | 6.1507 | 5.9958 | 4.8818 | 6.0052 | 6.6657 |
|  | 6 | 6.507 | 6.07 | 6.324 | 6.157 | 6.633 | 6.371 | 6.551 | 4.5492 | 4.4943 |
|  | 9 | 6.20 | 5.54 | 5.69 | 5.43 | 5.22 | 6.22 | 6.42 | 4.55 | 4.49 |
| Oleic - C18-1 cis | 0 | 19.34 | 21.28 | 21.85 | 19.02 | 19.54 | 19.01 | 18.93 | 18.11 | 18.98 |
|  | 3 | 18.5586 | 19.3736 | 17.5953 | 15.7984 | 18.4935 | 19.3083 | 15.922 | 21.0876 | 18.978 |
|  | 6 | 18.357 | 18.534 | 19.675 | 19.1 | 19.443 | 19.167 | 18.862 | 15.5311 | 16.0058 |
|  | 9 | 19.78 | 18.25 | 18.63 | 17.31 | 17.36 | 19.20 | 18.44 | 15.53 | 16.01 |
| Elaidic - C18-1 t | 0 | 0.57 | 0.44 | 0.60 | 0.51 | 0.59 | 0.53 | 0.52 | 0.63 | 0.65 |
|  | 3 | 0.532 | 0.6375 | 0.5514 | 0.6723 | 0.5998 | 0.5506 | 0.8313 | 0.6036 | 0.650 |
|  | 6 | 0.52 | 0.547 | 0.573 | 0.512 | 0.582 | 0.555 | 0.522 | 0.595 | 0.9647 |
|  | 9 | 0.58 | 0.63 | 0.56 | 0.69 | 0.64 | 0.67 | 0.60 | 0.60 | 0.96 |
| Linoleic - C18-2 cis | 0 | 15.88 | 16.54 | 19.94 | 15.10 | 16.76 | 17.26 | 16.97 | 21.85 | 17.17 |
|  | 3 | 16.3978 | 17.735 | 19.6433 | 21.5079 | 19.0358 | 18.53 | 28.5944 | 17.7542 | 17.174 |
|  | 6 | 16.633 | 17.54 | 18.063 | 18.33 | 17.959 | 19.093 | 20.553 | 25.2853 | 29.4956 |
|  | 9 | 20.40 | 23.84 | 20.68 | 27.45 | 25.96 | 21.51 | 22.49 | 25.29 | 29.50 |
| Arachidic - C20-0 | 0 | 1.14 | 1.18 | 1.28 | 0.97 | 1.23 | 1.28 | 1.18 | 1.71 | 1.03 |
|  | 3 | 1.0799 | 1.2676 | 1.3774 | 1.2601 | 1.3558 | 1.415 | 1.855 | 1.076 | 1.033 |
|  | 6 | 1.09 | 1.246 | 1.289 | 1.186 | 1.311 | 1.324 | 1.365 | 1.3921 | 1.746 |
|  | 9 | 1.30 | 1.54 | 1.39 | 1.58 | 1.61 | 1.44 | 1.47 | 1.39 | 1.47 |

**P**: Primary mixture, **P.T**: Primary mixture + turnip (8%), **P.Sq**: Primary mixture + butternut squash (8%), **P.S1**: Primary mixture + sourdough (0.5%), **P.S1.T**: Primary mixture + sourdough (0.5%) + turnip (8%), **P.S1.Sq**: Primary mixture + sourdough (0.5%) + butternut squash (8%), **P.S2**: Primary mixture + sourdough (1%), **P.S2.T**: Primary mixture + sourdough (1%) + turnip (8%), **P.S2.Sq**: Primary mixture + sourdough (1%) + butternut squash (8%)
